# Analysis of wastage mechanisms in the supply chain of fish products in a circular economy perspective: Empirical research

**DOI:** 10.1016/j.heliyon.2023.e17449

**Published:** 2023-06-19

**Authors:** Francesco Tola, Enrico Maria Mosconi, Giacomo Branca, Fabiana Natali, Mattia Gianvincenzi, Bogdana Nosova, Andrea Colantoni

**Affiliations:** a Department of Economics, Engineering, Society, Business Organization, University of Tuscia, Viterbo, Italy; b Department of Social Communications, Institute of Journalism, Taras Shevchenko National University of Kyiv, Kyiv, Ukraine; c Department of Agriculture and Forest Sciences, University of Tuscia, Viterbo, Italy

**Keywords:** Fish consumption, Food waste, Consumer behavior, Logistic regression, Culinary education

## Abstract

The problem of food waste at the post-consumption phase, as well as its rational management, is one of the central matters of the transition process undertaken by the European Union, and it is one of the priorities in the Action Plan for a Circular Economy. In particular, the waste of fish products occurs throughout its supply chain. A wide literature is provided in analyzing waste on the first part of the fishing supply chain while high importance is also given to its consumption phase, especially regarding dynamics, causes and social behaviors. The paper aims at investigating mechanisms and causes of wastage that occurs in the last part of the supply chain, at a local level in Italy, by focusing the analysis at the final stage of fish consumption, produced by Italian households. Among the causes, the change in consumption habits and attitudes is also influenced by the restrictions of the healthy regulations adopted for the pandemic. Specifically, a survey was conducted to investigate what actions, habits and behaviors contribute to producing fish food waste. Causes of waste have been discussed by framing them under two dimensions of final consumption, home consumption and out-of-home consumption. Findings carried out from the research suggest knowledge of channels of distribution and integrative elements to manage the leftover food as well as the intensity of fish consumption are basilar to understand the wastage mechanism in a circular perspective. This study is an assessment of whether factors identified as causes, may increase, or decrease the waste. The importance of realizing explorative studies at the consumption phase under the approach of considering fish waste, not only as a fraction into the food waste but even a distinct part as a whole, is also given by the need to create specific strategies for sustainable production and consumption. A reflection is conducted as to how this study can be the basis for future studies on other food products related to the challenge of the rational management of food waste studies in a circular perspective.

## 1 Introduction

Food waste is defined as “food appropriate for human consumption that is discarded or left to deteriorate at the consumer level regardless of the cause” [1]. It has become a relevant topic in recent years due to the economic and environmental burdens it entails. Fishery products are perishable foods that have a high potential for waste and loss. Their consumption reached 20.5 kg per capita per year in European countries. In Italy, the average fish household consumption reaches 23 kg per capita [2]. It is estimated a future growth due several factors including market and lifestyle changes [3,4]. At a worldwide level, the waste of fishery products is a part of a more general problem of the sector that is linked to United Nations Sustainable Development Goal indicator number 12 (SDG 12). In fact, it is also estimated that for every year, about 1/3 of the food produced becomes waste. It is a volume equivalent to about 1.3 billion tons of wasted food [5]. The United Nations (UN) has set the objective of halving the world food waste as part of its program of 2030 Agenda [6], also by reducing global food loss (SDGs 12.3). Under the SDG perspective, the specific problem of fish waste is managed into the indicator 12.3 by FAO and UNEP environments. The indicator, in turn, is divided into two sub-indicators in that different drivers along the food chain generate food waste and losses. Specifically sub-indicator 12.3.1b monitors and measures waste in the last stages of the supply chain. The measurement methods can be different: questionnaires, interviews, surveys, statistical coefficients, and agendas are the most widespread in the scientific field [7]. According to the latest revision, the sub-indicator is classified as tier 2 [8]. At the European level the circular economy strategy for food waste focuses on key priority actions within the “Farm to Fork” Program (Farm to Fork Strategy for a Fair, Healthy, and environmentally Friendly System, n.d.). In this sense, quantifying food waste is a starting point, essential for the development of effective prevention and reduction strategies and for verifying their effectiveness over time [9,10]. About 931 million tons of food waste were generated in 2019 [7], 61% of which came from households, 26% from catering and 13% from retail. Similarly, the EU produces 88 million tons of food waste and only households generate more than half, 47 million tons, followed by restaurant services and retail trade [11]. Different types of fish waste are generated along the supply chain, both at sea and on shore during fish processing, sale, and consumption. Specifically, a huge part of the losses occurs during fishing when unwanted fish or parts of a fish caught are discarded back into the oceans [12]. However, a great part is wasted at the retail store and household levels. Although the consumption stage presents a relatively high level of this problem the wide literature focuses mainly on discussing causes related to the first parts of the food supply chain. Consumer attitudes towards eating fish play an important role in explaining people’s behaviors in relation to production of fish food waste. Perceptions of fish as a healthy dietary choice, involvement with fish, wider interest in healthy eating, taste, and consumer lifestyles are important elements that affects consumptions and waste production [13,14]. Wastage is strongly linked to the food-related lifestyle studies that are important determinant for food-related behaviors. They support the study of consumers as active players for causes of waste. It is argued that consumers' attitudes towards preventing waste, perception of control, and norms can affect their intentions to avoid fish food waste; their intention, in turn, may affect wasteful behavior [15].

The paper is structured as follows. The first section addresses the background of the study. It is followed by details regarding the methods employed, including the clustering analysis that used a probabilistic approach. The results of our empirical study are then presented. Finally, the main findings are discussed, within the result and discussion section.

## 2 Literature review

Since consumers are responsible for a high percentage of the total food waste, its reduction, at the household level, is considered one of the most important actions necessary to improve the quality of the environment on par with the food safety [16].

A wide literature analysis addresses the complexity of the interdependent factors and behaviors that affect food waste generation. Many conceptual frameworks have been developed in recent years. The literature agrees that causes are diverse and not always obvious [17]. However growing studies on identifying the main factors for consumer-related food waste such as the motivation to avoid them, consumers' food managing capabilities and the handling of trade-offs between priorities has proven that customer behavior is an important element of study in this field [18]. It is well known that quantity and intensity of willingness to buy food are one of the main causes of food presence in domestic waste [19]. Preparing too much food can lead to increased food waste at home [20]; but consumer preferences such as taste, freshness, appearance, and nutritional quality play a relevant role in food waste generation [21]. For example, [22] the good sensory capabilities of the product help the consumer in determining the freshness of the food to minimize or prevent waste. Alongside appearance, taste is a powerful barrier to their reduction, particularly for consumers who believe eating should be pleasurable and consider it unthinkable and undesirable to eat distasteful foods simply to reduce food waste [23].

On the other hand, studies examining the goals of consumers have provided initial evidence to suggest that food-handling practices might be influenced by motivational factors. When handling food, consumers are likely to assign importance to multiple goals, including that of being a good provider (i.e., providing plenty of healthy and tasty food to children, guests, or partners) [24]. Specific aspects such as overcooking during the preparation of food, seem must be included among the main factors of food waste [25]. At the upstream, it could be that abnormally shaped products require handling or cooking skills that consumers may not have [26]. The education to re-use reuse by consumers can also be used to reduce them at home [27].

An important role is played by the household demographics. Demographic factors, including education level and gender, attitudinal and behavioral patterns related to food waste and the degree of concern regarding the issue, have also been identified as influencing food-waste behaviors [28]. Large households [29], as well as those with a high-income impact food-waste behaviors [30]. Finally, at the consumer level, age appears to be negatively correlated with wasted food quantities. Young adults are one of the highest-wasting groups [31]. Restaurants and food services represent a significant proportion of food waste [32]. Ref. [33] suggest critical factors at the consumption phase such as the research of satiety for the customer by the mean of portion sizes are among the main causes in the catering sector. Food waste produced outside the home can be limited by embodying applications that allow the management of leftover food such as the doggy-bag service [34]. Furthermore, changes in consumer preferences towards specific modes of food shopping, such as online versus offline [35] have been studied also considering the effect of the pandemic period, especially on how people buy, prepare and store food [36,37]. It is widely proven, the Covid-19 pandemic has significantly impacted food consumption patterns especially in the catering sector [38].

All the literature converges in arguing that to be aware of the reduction mechanism of consumer-related food waste, it is important to open to an in-depth understanding of the factors shaping food waste-related consumer perception and behavior, both in the household as well as the outside the home. Knowledge of food waste involvement is understood as the level of awareness and concern about the issues related to the phenomenon that have a proven effect on wasting behavior [39]. People who are aware of the problem of food waste are more likely to avoid wasting food.

This study aims to analyze the creation of food waste from fish products by Italian families. Behaviors and reasons of consumers both inside and outside the habitation are also considered to study the mechanism behind waste production. In this sense a survey is presented as the first research approach aimed at quantifying fish waste for families, differentiating the waste produced inside and outside the home also in light of the consideration of the current pandemic crisis. Concerning the last aspect analyses were carried out considering changes provided by the safety regulation for commercial and catering activities such as various closures for the containment of the virus. The scenario of the current pandemic crisis was observed, examining whether the change in consumption of families in taking advantage of home delivery of fish-based dishes, has changed the volume of waste at home. In this way, one of the goals of the study is to investigate whether the frequencies of home deliveries have contributed to increasing the fish products HW.

The literature review and background inspired the following research questions:

RQ1) What are the causes that determine the food waste of fish products at a household level?
RQ2) How consumer attitudes play a role in people’s behaviors. How channels of distribution and integrative elements to manage the leftover food relate to the wastage mechanism?
RQ3) Did, door-to-door delivery, during the Covid-19 pandemic containment, influence the production of household waste of fishery products?

## 3 Material and method

In order to answer the research questions, an online cross-sectional survey was conducted to collect data, based on the background analysis and review, also on survey approaches, referred to food waste and quantification methods for household food wastes [28,[40], [41], [42], [43]]. Questionnaires focus on a sampling of a smaller group of people that are statistically representative of the wider population of the local area of Civitavecchia and Viterbo Cities in Italy. The survey was made available by mean of social and multimedia platforms. A total of 214 families filled out the online questionnaire, from the area between the two cities. Basically, the survey conducted, and its results are part of a broader research aimed at studying consumer behavior in specific categories of food through different types of purchase (take-away, restaurants, retail, etc.). The survey, consists of 24 questions in open and multiple-choice format, divided into four areas. Every variable described in the questionnaire, and subsequently elaborated within the Logit model, is defined under a specific literature reference on consumption related to food waste causes for both household and out-house dimension. They are synthetically shown in the table below (Table 1).

**Table 1:** Literature references for the variables.

Independent Variable	Specific literature reference
Level-wage	[44,45]
Age	[29,46]
Frequency of consumption	[28,47]
Purchasing Responsibility	[48]
Frequency of takeaway	[49]
Food Waste awareness	[39]
Excessive purchases	[50,51]
Malodor	[20]
Different taste	[52]
Incorrect preservation	[15]
Reuse waste	[53]
Satiety	[42]
Wrong meal	[54]
Leftover application	[24,34]

The first section of the questionnaire investigates on domestic consumption, purchase preferences, trends, and habits in fish product purchasing. The second section focuses to extract on the frequency of out-of-home consumption of fish products and the frequency of recourse to takeaway also during the closing pandemic period. The third section concerns questions on the food waste awareness, starting from the definition proposed by Ref. [1], and the self-perception of the waste of fish products both HW and NHW, associating the causes for which the waste occurred. For the quantification of fish waste produced as HW and NHW, it was asked to self-declare the waste threshold in a numerical range from 0 to 100. The technique used for the quantification has already been tested in other studies dealing with food waste in the last stages of the supply chain. The fourth section is regarding socio-demographic questions.

### 3.1 Data collection and analysis

The survey was made available by the mean of social and multimedia platforms. A total of 214 families filled out the online questionnaire, from the area between Viterbo and Civitavecchia cities.

The collected data were entered into a database and modelled through the conjunct manual work of the authors for the dichotomization of the two dependent variables (FWHOUSEHOLD-FWNONHOUSE). Once the database was created, the data was transferred to the Gretel software for processing the survey responses.

Two logistic regression models were used through the Gretel software to identify the factors and causes that generate fish waste. The Logit model is mainly used in the social sciences [55], describing the probability of a relationship between a dichotomous variable and one or more quantitative or qualitative variables. The Logit model is a widely used methodology in the literature for estimating the probability of increasing or reducing food waste according to various factors [34,53,[56], [57], [58]].

Specifically, the two logistic regression models were estimated for the self-reported quantification of HW and NHW. The dichotomous dependent variable of HW of fish product is defined through the indications of [27], suggesting the threshold value for waste estimated through the arithmetic mean of the self-declared value of the sample, corresponding to χ = 14.5. The dichotomous dependent variable of NHW of fish product was estimated from the study [34], which identify waste in restaurants if the self-reported value is five or higher. Subsequently, the independent variables were selected to build the probabilistic regression models focused on the quantification of HW, NHW, and consumer behavior. The frequency of consumption [20], the person responsible for the purchases and the knowledge of the impacts on food waste [59] are suggested as independent variables for HW, while the most frequent and consolidated causes are extrapolated from previous studies and the reuse has been considered the best method to reduce waste [60]. The takeaway consumption frequency has been inserted to seek the change in consumer behavior due to the closure of the restaurant activities, which has led to a change in waste generation. The frequency of consumption at the restaurant [61] and the knowledge of the waste impacts are suggested as dependent variables for NHW, while the possible causes are indicated by Ref. [34], and the doggy-bag is identified as the method to reduce waste [45].

## 4 Result and discussion

Among the socio-demographic characteristics of the sample, collected through the surveys analysis and listed in Table 2, emerges that almost two-thirds of the participants are composed of women, with an average age standing at 41.62 years ranging from 18 to 85 years old. Almost 4 out of 5 of the participants are not located in a coastal area and have no children under 12 years of age. More than half of the sample has a university-level education and belongs to the middle level of the income ranking.

**Table 2:** Characteristics of the population.

FEATURES	DISTRIBUTION OF THE SAMPLE
Gender	102- Women (63.35%) 59- Men (36.65%)
Average age (range)	41.62 (from 18 to 85 years old)
Residence area	35- Coastal area (21.74%) 126- Inland area (78.26%)
Nuclear family	33- Presence of children up to 12 years (20.50%) 128- No children (70.50%)
Education level	6- Primary education (3.73%) 72- Secondary education (44.72%) 83- Higher education (51.55%)
Monthly wage	53- first income bracket 0 € - 1000 € (32.92%) 91- second income bracket € 1001 - € 2500 (56.53%) 17- Third income bracket> € 2501 (10.55%)

The descriptive statistics of the dependent variable FWHOUSEHOLD are reported in Table 3. The methodology applied for the dichotomization of the FWHOUSEHOLD variable revealed that, in the sample, 36,65% of the families declared a value above the average, while the remaining 63,35% assigned a value below the average.

**Table 3:** Descriptive statistics FWHOUSEHOLD.

“FW HOUSEHOLD”	
Average	14.460
Median	10,000
Meansquaredeviation	16.081
Min	0.00
Max	70.00
Coefficient of variation	1.1122

On the other hand, Table 4 provides statistical indicators for the dependent variable NHW. Such values show opposite results with respect to HW variables. In fact, the dichotomization of the variable NHH reveals that 68.94% of the families reported more than the laid down limit for waste production (5%) consuming fish outside the house. The remaining 31.06% of those surveyed declared a lower value. In addition to the averages of the two descriptive statistics, it is confirmed that less waste occurs at the household level (14.460%) than outside (16.950%).

**Table 4:** Descriptive statistic FWNONHOUSE.

“FW NONHOUSE”	
Average	16.950
Median	10,000
Meansquaredeviation	19.161
Min	0.00
Max	70.00
Coefficient of variation	1.1304

The results of the logistic regression model of the factors affecting the significance of the dichotomous variable FWHOUSEHOLD are shown in Table 5.

Considering the value of Mcfadden’s R-square is equal to 0.358169 underlined (see Table 5). The value is in line with scientific studies on food waste through a self-declaration methodology of waste with qualitative variables [57,62].

**Table 5:** Logit model FWHOUSEHOLD.

FW HOUSEHOLD					
	*Coefficient*	*Std.error*	*z*	*p-value*	
Const	1.29661	1.47908	0.8766	0.3807	
Children	0.431740	0.585428	0.7375	0.4608	
Level-wage	1.03305	0.521747	1980	0.0477	**
Education	−0.486061	0.513139	−0.9472	0.3435	
Residence	0.320491	0.642138	0.4991	0.6177	
Age	−0.0701882	0.0200108	−3.508	0.0005	***
Gender	0.417618	0.522192	0.7997	0.4239	
Frequency-of-consumption	1.83718	0.534871	3.435	0.0006	***
Purchasing-responsibility	−0.889995	0.551833	−1.613	0.1068	**
Low-frequency-of-takeaway	0.586563	0.612318	0.9579	0.3381	
Medium-frequency-of-takeaway	1.49369	0.656277	2.276	0.0228	**
High-frequency-of-takeaway	−1.55592	1.20612	−1.290	0.1970	
Food-Waste-awareness	−1.95638	0.804838	−2.431	0.0151	**
Excessive-purchases	0.635325	0.305759	2.078	0.0377	**
Excessive-culinary-preparations	−0.280190	0.305424	−0.9174	0.3589	
Malodor	0.556362	0.279995	1.987	0.0469	**
Product-expiration	−0.213339	0.263461	−0.8098	0.4181	
Visceral-parts	−0.141371	0.235481	−0.6003	0.5483	
Different-taste	0.603534	0.264178	2.285	0.0223	**
Incorrect-preservation	−0.500266	0.231820	−2.158	0.0309	**
Reuse-waste	−0.220398	0.173604	−1.270	0.2042	*
McFadden’s R-framework				0.358169	
Number of cases ‘predicted correctly’				132 (82.0%)	

Among the influencing factors, there is the *level-wage*. The model returns the coefficient of the variable with a positive value, at a significance level of α = 0.05, which means that families with higher incomes, having more purchasing power, may generate greater fish products HW. This result confirms studies by Refs. [44,45], which agree that people with higher wages waste more than people with lower wages.

Another important factor is the *Age*. With a negative value and a significance level of α = 0.01, is interpreted as young people probably producing more fish products HW than adults, in line with the studies of [29,46], due to a multitude of factors ranging from the lower degree of maturity, environmental sensitivity and economic management skills.

The *frequency-of-consumption* returned a positive coefficient at a significance level of α = 0.01. Thus, an increased purchasing intensity, understood as increased frequency of consumption, has the probability to affect the amount of waste generated HW. This hypothesis may depend on several factors, such as behavioral norms, social norms, and procurement practices.

With the regard the *purchasing-responsibility* coefficient the score is also positive. A significance level of α: 0.05, confirms the studies of [48] stating that involvement and participation in the purchase phase help to decrease the food waste produced at home.

During the current pandemic crisis, home delivery services by catering businesses have grown. The *never-takeaway* variable was omitted from the model in the response phase of the results. The model returns the coefficient of the frequency of take away variable with a positive value, at a significance level of α: 0.05. The results show that increasing the frequency of home delivery, as well as the frequency of consumption, can probably generate an increase in food waste produced [49].

The model returns the coefficient of the *food-waste-awareness* variable with a negative value, at a significance level of α = 0.05. The knowledge of food waste and the involvement of food waste as the level of awareness and concern of the problems related to the phenomenon have a proven effect on waste production behaviors, in Ref. [39] it’s believed that people who are aware of the problem of food waste are more inclined to avoid of wasting food and responsible. Subsequent influencing factors discern from it, such as excessive purchases, malodor, different taste, incorrect preservation, and reuse.

The excessive purchase is one of the main causes of food HW [39]. The model returns the coefficient of the *excessive-purchases* variable with a positive value, at a significance level of α = 0.05, therefore, higher purchases correspond to higher possibilities to generate fish product HW. The model’s response agrees with studies by Refs. [50,51], which stated buying more food than necessary increases the likelihood of waste.

The product must have good sensory capabilities because they help families determine the freshness of the food to minimize or prevent waste [20]. For this reason, the coefficient of the *malodor* variable results positive with a significance level of α: 0.10, which means a fish product with a bad smell has more chance to become a waste.

The sense of taste, as well as smell, also needs to be satisfied. The coefficient of the *different-taste* variable has a positive value with a significant level of α: 0.05, this result shows that variations in taste compared to expectations increase the probability of generating waste [52].

Smell and taste can be disregarded due to incorrect conservation, which however, in the model analyzed, does not affect the generation of waste. This result is interpreted as the families in the sample have a good knowledge of the method of conservation of the various fish products.

The best solution against fish product HW is reuse and it is confirmed by the model. The coefficient of the *reuse* variable has a negative value at a significance level α = 0.10, so people who do not reuse fish product waste are more likely to waste. Ref. [53] showed that individuals who do not recycle or compost any of their kitchen waste tend to throw away more food than those who reuse kitchen waste.

The results of the logistic regression model of the factors affecting the significance of the dichotomous variable FWNONHOUSE are shown in Table 6.

**Table 6:** Logit model FWNONHOUSE.

FW NONHOUSE					
	*Coefficient*	*Std.error*	*z*	*p-value*	
const	−0.365402	1.52817	−0.2391	0.8110	
Children	0.0938731	0.575660	0.1631	0.8705	
Level-wage	0.0885697	0.461335	0.1920	0.8478	
Education	0.115966	0.440648	0.2632	0.7924	
Residence	−0.788242	0.516659	−1.526	0.1271	
Age	−0.00972016	0.0148706	−0.6536	0.5133	
Gender	0.0894448	0.437055	0.2047	0.8378	
Frequency-to-the-restaurant	0.0541500	0.932143	0.05809	0.9537	
Food-Waste-awareness	1.22905	0.732953	1.677	0.0936	*
Satiety	0.429844	0.192167	2.237	0.0253	**
Different-expectations	0.0881395	0.198227	0.4446	0.6566	
Wrong-meal	−0.521014	0.238497	−2.185	0.0289	**
Fish-cleaning	0.490758	0.213059	2.303	0.0213	**
Covered	0.332175	0.242696	1369	0.1711	
Doggy-bag	−0.291299	0.149629	−1.947	0.0516	*
McFadden’s R-framework				0.200312	
Number of cases ‘predicted correctly’				132 (82.0%)	

The value of the goodness of fit of Logit model FWNONHOUSE is determined by McFadden’s R-square, which corresponds to 0.200312underlinedand it is comparable to studies of [22]. The results are the following:

Ref. [42] affirmed that satiety due to portion sizes is a major cause of food waste in the catering industry. In line with this study, the model demonstrates that the coefficient of the *satiety* variable is positive with a significance level of α: 0.01, proving that the higher an individual’s level of satiety, the greater his relative waste.

Wrong communication in making orders to the waiter, as well as the bad composition of a dish, can increase food waste [54]. The model returns the *wrong-meal* variable with a negative coefficient value at a significance level of α: 0.05. Therefore, despite errors in the communication or assembly phase of the order, the model demonstrates that the likelihood of generating fish products NHW does not increase.

The model demonstrates that the coefficient of the *fish-cleaning* variable is positive at a significance level of α: 0.05. Accordingly, the probability to generate more fish product NHW when the courses are served with the presence of shells, bones, or carapace is confirmed.

Initiatives for reusing fish leftovers is a good way to reduce food waste in the catering sector. The coefficient of the *doggy-bag* variable is negative at a significance level of α: 0.1. In line with the study by Ref. [24], which shows that not requiring the doggy-bag for the leftovers has a greater chance of generating waste.

### 4.1 Limitation of research

On one side, the guidelines for the quantification of food waste [7] propose to use the survey as a method to deepen a qualitative viewpoint and enrich the research with additional information. On the other side, the European Commission proposes to combine the survey with a methodology aimed at increasing reliability and relevance. In contrast to the limits of research, [63] considers that surveys are structured and formal means of collecting quantitative and qualitative data between all actors in the agri-food chain, from producers to consumers; they are affordable, usually standardized, accessible, and easy to read.

## 5 Conclusion

Food waste is made up of different elements which have also different properties and thus possible different paths. This study represents a starting point for the analysis of the mechanisms that affects the fish waste production at the consumption phase. In General, the results show a great importance to the culinary knowledge and a better awareness during the preparation phase of food. The age factor outlines that young people is more sensitive to these variables, playing, them, a relevant role in producing food waste. Concerning the household waste behaviors such as: a more attention to the shopping list composition, careful selection of the product also by checking label, shelf-life information has the power to limit the probability of fish waste. It is interesting to observe that purchase frequency for families is not inversely proportional to the wastage production. The pandemic crisis has influenced consumption behavior. The recourse to the take-away frequency has been analyzed as a possible cause of wastage. Specifically, the direct proportionality of the frequency and range of waste suggest that a more attentive and informed consumer is crucial to reducing waste.

Overall, the model of fish product HW returns three significant variables, which deserve particular attention, the different taste compared to expectations and malodor, that increase the chance to generate waste, and the practice of reusing food waste from fish products, which is lacking in family habits.

Information on organoleptic properties of the various fish products can act on “not like taste” manifestations and more in general, on an increased of the personal taste expectations [63]. It is valid especially for household Wastes. Is not to be underestimated the “bad smell” factor, that is also a sign of needing for good practices to be adopted to prevent fish wastes.

In this sense, according to the literature, the use of communication channels showing effective practices, and new culinary recipes including the reuse of fish product waste, praising its advantages (i.e., economic savings), could help in managing the problem [64].

From the analysis, also the purchasing in excessive quantities is a key factor influencing the fish product HW, which can be attributed to incorrect planning in the purchase phase or to incorrect communication within the family unit. Paying more attention in the purchase planning phase or checking inventory can reduce the excess of fish products purchased and consequently reduce their waste.

The status of gutted or ungutted fish is an important variable to consider for the two dimensions of consumption (HW and NHW) as well as practices in cleaning fish product, perceived differently according to the place of consumption.

The offer an additional service to the consumers aimed to increase the awareness of the techniques for reusing fish leftovers, also by the mean of digital application, is another factor of optimization for fish-food waste. Guidelines and recommendations with rewards to the access are growing tools used for the grocery and Horeca sectors.

The portion size and the use of the doggy-bag are the significant variables in the fish products NHW. From the survey emerged that the sense of satiety affects the variable NHW, determining the probability of generating waste as the sense of satiety increases due to the portions of fish-based dishes not aligned with the consumer needs. Activities involving [51] the restaurateur and the customer, i.e., inserting the size of portions in the menu, have the potential to affect and manage indirectly the amount of waste and at the same time offer cost-saving alternative. A factor that can contribute significantly to reducing the fish product NHW are leftover applications such as the doggy-bag. Ref. [65] showed the large-scale adoption of doggy-bags requires an increase in the perceived benefits and a reduction of negative feelings (i.e., sense of shame associated with in recovering leftovers) of the consumers. To implement the doggy-bag strategy, public bodies should contribute to being the guarantor of the education and prevention campaigns of food waste, encouraging its use and increasing its practicality, reversing negative thoughts into proactive ones, and encouraging catering activities that practice the spread of use.

The importance of the approach in considering fish waste not only as a fraction of food waste but as a distinct element as part of a whole represents the groundwork for a deeper exploration of this topic and provides an opportunity to replicate the study for other different food waste components.

From a circular economy perspective, the reuse of products in the culinary sector appears to be the most popular practice in limiting waste.

Thus, results of the study may support future research aimed at developing strategies to mitigate fish waste generated during the consumption phase. The study provides better knowledge on fish waste generation drivers, concerning the Italian families, both for household and non-household consumption, paving the way for new studies that focus on the waste of a certain food. The search can also be applicable in other regions.

## Author contribution statement

Francesco Tola, Fabiana Natali: Conceived and designed the experiments.

Francesco Tola, Enrico Maria Mosconi and Fabiana Natali: Performed the experiments.

Francesco Tola, Fabiana Natali and Mattia Gianvincenzi: Analyzed and interpreted the data.

Francesco Tola, Enrico Maria Mosconi, Giacomo Branca, Fabiana Natali and Bogdana Nosova: Contributed reagents, materials, analysis tools or data.

Francesco Tola, Andrea Colantoni, Mattia Gianvincenzi, Fabiana Natali, Giacomo Branca, Enrico Maria Mosconi and Bogdana Nosova: Wrote the paper.

## Data availability statement

Data included in article/supp. material/referenced in article.

## Declaration of competing interest

The authors declare that they have no known competing financial interests or personal relationships that could have appeared to influence the work reported in this paper
