# Supplementary material for: Analysis of wastage mechanisms in the supply chain of fish products in a circular economy perspective: Empirical research
Source: Heliyon. 2023 Jun 19;9(6):e17449. doi: 10.1016/j.heliyon.2023.e17449 (PMC10333618; doi:10.1016/j.heliyon.2023.e17449)
Supplement: Multimedia component 1 [file mmc1.docx]

**confirming that informed consent was obtained from all participants for your research.**

**1. How often do you eat fish products? ***

- Four or more times a week
- Two or three times a week
- Once a week
- Less than once a week
- I don't eat fish

**2. On a scale of 1 (very low) to 5 (very high), please tell me your liking for the following categories of seafood.** *

- Blue or white fish (sea bream, sea bass, red snapper, mullet, tuna, hake, sardine, salmon)

1-2-3-4-5

- Crustaceans (shrimp, scampi, lobster, crab)

1-2-3-4-5

- Molluscs (clams, mussels, dates, octopus, limpet)

1-2-3-4-5

**3. Who in your family is responsible for purchasing seafood? ***

- Me
- Me, along with another family member
- Another member of my family

**4. Where do you usually buy fish products? ***

- Supermarket
- Market
- Fishmonger
- Directly from the fishermen
- Other:

**5. On a scale of 1 (not at all) to 5 (very much), indicate which of the following factors affect your seafood purchase the most. ***

|  | 1 | 2 | 3 | 4 | 5 |
| --- | --- | --- | --- | --- | --- |
| Local origin |  |  |  |  |  |
| Nutritional properties |  |  |  |  |  |
| Freshness |  |  |  |  |  |
| Recurrences / traditions (e.g. Eve, New Year's Eve) |  |  |  |  |  |
| Transformation / processing (e.g. product already filleted, product in oil) |  |  |  |  |  |
| Sustainable capture method |  |  |  |  |  |
| Price |  |  |  |  |  |
| Presence of offers at the point of sale |  |  |  |  |  |

**6. On a scale of 1 (never) to 5 (always), indicate how often you take the following actions before and during the seafood purchase phase. ***

|  | 1 | 2 | 3 | 4 | 5 |
| --- | --- | --- | --- | --- | --- |
| I systematically look at the labels of the various products |  |  |  |  |  |
| I use a shopping list |  |  |  |  |  |
| I plan future meals before my purchases |  |  |  |  |  |
| Purchase by expiration date |  |  |  |  |  |
| Purchase based on the quantities indicated in the recipes |  |  |  |  |  |

**7.Covid has changed some habits, including eating out. How often did you eat seafood outside the home before the pandemic? ***

- Four or more times a week
- Two or three times a week
- Once a week
- Less than once a week
- Once or twice a month
- Less than once a month
- Never

**8.During the periods of closure to the public of the catering activities, have you ever taken advantage of the delivery of fish products already prepared? (ex: sushi, pasta, soups, etc. )***

- Yes
- No

**9. How often did you use home delivery / takeaway of fish products?** *

- Four or more times a week
- Two or three times a week
- Once a week
- Less than once a week
- Once or twice a month
- Less than once a month
- I have never used it

**10. Have you ever heard of the phenomenon of food waste? ***

- Yes
- No

**11. Answer yes or no to the following questions:** *

|  | YES | NO |
| --- | --- | --- |
| I think that food waste is interconnected with human health and the environment |  |  |
| I think food waste only occurs in non-industrialized countries |  |  |
| I think the environmental impact of food waste is minimal in industrialized countries |  |  |

**12. From your point of view, what aspects do you think food waste can affect** *

|  | YES | NO |
| --- | --- | --- |
| Waste of money |  |  |
| Waste of time |  |  |
| Waste of energy and electricity |  |  |
| Waste of water resources |  |  |
| Increase in waste produced |  |  |

**13. Please indicate in what percentage (values from 0 to 100) you think you are wasting seafood when you eat at home ***

**14. On a scale of 1 (not at all) to 5 (very much), it indicates how much the following factors affect the increase in the waste of fish products at home**

|  | 1 | 2 | 3 | 4 | 5 |
| --- | --- | --- | --- | --- | --- |
| I bought too much food |  |  |  |  |  |
| I cooked more than necessary |  |  |  |  |  |
| The product has a bad smell |  |  |  |  |  |
| The product has passed the expiration date |  |  |  |  |  |
| The product has visceral parts |  |  |  |  |  |
| The taste does not reflect my expectations |  |  |  |  |  |
| I have not stored the product properly |  |  |  |  |  |

**15. On a scale from 1 (never) to 5 (always), it indicates how often you reuse fish waste (head, shell, carapace ...) for subsequent preparations (sauces, fumet ...) ***

| 1 | 2 | 3 | 4 | 5 |
| --- | --- | --- | --- | --- |

**16. Please indicate in what percentage (values from 0 to 100) you think you are wasting fish products when you eat away from home (eg at a restaurant) ***

17. On a scale of 1 (not at all) to 5 (very much), it indicates how much the following factors affect the increased waste of seafood outside the home. (eg at the restaurant).

|  | 1 | 2 | 3 | 4 | 5 |
| --- | --- | --- | --- | --- | --- |
| The dishes ordered fill me up before I can finish them |  |  |  |  |  |
| The dishes have an unwelcome taste |  |  |  |  |  |
| The dishes show defects with respect to the order |  |  |  |  |  |
| The dishes are difficult to clean (it is difficult to remove the shell or the bones) |  |  |  |  |  |
| The cover charge (eg bread, bread sticks, focaccia) makes me full before the dishes arrive |  |  |  |  |  |

**18. On a scale of 1 (never) to 5 (always) it indicates how often you ask to take any leftover fish products home ( doggy-bag ) at the end of a meal away from home (restaurant) ***

| 1 | 2 | 3 | 4 | 5 |
| --- | --- | --- | --- | --- |

**19. Age ***

Please indicate only the number of your years

**20. Gender** *

- Woman
- Man

**21. Educational level** *

- Primary or middle school license
- High school diploma
- Degree
- Master / Doctorate

**22. Area of residence** *

- Coastal area
- Internal area

**23. Occupation ***

- Student
- Busy
- Household / a
- Unemployed / job seeker
- Retired / a

**24. Which of the following categories does your monthly income fit into?** *

- 0 € - 1000 €
- € 1001 - € 2500
- € 2501
- Rather not answer

**25. Are there children aged 12 or under in your household ?***

- Yes
- No
